# Effect of *Aegle marmelos* and *Murraya koenigii* in treatment of delayed pubertal buffaloes heifers

**DOI:** 10.14202/vetworld.2016.1375-1380

**Published:** 2016-12-06

**Authors:** Mohan M. Baitule, A. P. Gawande, Umesh Kumar, S. K. Sahatpure, Manoj S. Patil, Mansi M. Baitule

**Affiliations:** 1 Department of Animal Reproduction, Gynaecology and Obstetrics, Nagpur Veterinary College, Maharashtra Animal & Fishery Sciences University, Nagpur - 440 006, Maharashtra, India; 2 Department of Biochemistry, Hislop College, Nagpur - 440 001, Maharashtra, India

**Keywords:** *Aegle marmelos*, buffaloes heifer, conception rate, *Murraya koenigii*, estrus, ovulation

## Abstract

**Aim:**

This study aims to study the estrus induction, ovulation, and conception rate of delayed puberty in buffaloes heifers by feeding a herbal plants *Aegle marmelos* (bael/bili/bhel leaf) and *Murraya koenigii* (Curry leaf).

**Materials and Methods:**

Totally, 24 buffalo heifers with delayed puberty were selected for the present study and divided randomly in four equal groups (n=6). Before experiment, all animals were dewormed with albendazole at 10 mg/kg body weight to prevent them from the stress of parasitism. In the present experiment, four group taken and Group I (n=6) treated with *A. marmelos*, Group II (n=6) treated with *M. koenigii*, Group III (n=6) treated with mixture of *A. marmelos* and *M. koenigii* and fed for 9 days. Group IV (n=6) considered as control and fed with concentrate only. The blood samples were collected from all the animals on day 0 (before treatment), 4, 9 (during treatment), on the day of estrus and day 8 after the onset of estrus. The 10 ml blood was collected from the jugular vein of all the experimental animals for estimation of serum calcium, inorganic phosphorus, and progesterone (P_4_). The estrus response, ovulation, conception rate along with serum calcium, inorganic phosphorus, and progesterone level were determined by the standard protocol.

**Results:**

From Group III 4 heifers, from Group II 3 heifers, and from Group I and IV (Control) 2 heifers each, exhibited the estrus. The estrus response was recorded as 33.33%, 50.00%, 75.00%, and 33.33% in Group I, Group II, Group III, and Group IV, respectively. In treatment Group III, serum calcium found significantly more (p<0.05) on day 8 post-estrus as compared to other groups at a similar interval. Inorganic phosphorus and progesterone show no significant difference between groups. The ovulation and conception rates are comparatively better in Group III (75%) buffalo heifers than other groups.

**Conclusion:**

Herbal supplementation of *A. marmelos* and *M. koenigii* in combination, as well as M. koenigii alone, were found effective in fertility improvement in delayed pubertal buffalo heifers by increasing ovulation and conception rate.

## Introduction

Livestock rearing is one of the most important economic activities in the rural areas as it provides supplementary income for most of the families dependent on agriculture. More than 67% of dairy animals are owned by marginal and small farmers. Interestingly, buffalo milk accounts for the significant share of the total milk production in the country and plays an important role in livestock economy of India.

The milk production is dependent on the reproductive status of the animals. Various reproductive disorders alter the productive potential of the animals. The incidence of pubertal anestrus in buffalo heifers is 56%, and in adult buffalo, it is 32.38%. The delayed puberty increases the age at first calving and thus it also reduces the total numbers of lactations and offspring.

Various varieties both hormonal and non-hormonal in nature are normally used for correcting the reproductive problems. Hormonal preparations are delivering good results, however, the high costs and chances of side effects always remain a speculation. Many herbal preparations are being marketed by the Indian Pharmaceuticals such as Prajana, Janova, Aloes compound, Sajani, and Heatquick, are used to correct delayed puberty, post-partum anestrus. These formulations are patent combination of herbs used to induce ovarian activity.

India is the reservoir of large number of medicinal plants. Since ancient time the plants of medicinal values are being used for the treatment of various diseases and disorders of human as well as animals. *Aegle* *marmelos* and *Murraya* *koenigii*, the medicinal plants are known for their antioxidant property. Van Miert [1] studied the effect of *A. marmelos* and *M. koenigii* by feeding the dried leaves powder in delayed pubertal buffalo heifers and reported remarkable estrus induction response. However, more experimentation is needed so that the use of these plants can be recommended in reproductive disorders of animals. *A. marmelos* and *M. koenigii* are easily available plants and one being used in our day to day life and could be an economical source.

Therefore, the present study was undertaken to evaluate effect of *A. marmelos* and *M. koenigii* on estrus induction, ovulation, and conception rate in delayed pubertal buffalo heifers.

## Materials and Methods

### Ethical approval

The experiment followed the guidelines of Institutional Animal Ethics Committee, and experiments were conducted in such a way that cruelty to the animals was minimized to the minimum.

### Sampling

The present study was conducted to assess the effect of *A. marmelos* and *M. koenigii* in the treatment of delayed puberty in buffaloes heifers in and around Nagpur district. It was ensured that all the experimental animals were maintained with standard managemental practices during the experiment. Before the experiment, all animals were dewormed with albendazole at 10 mg/kg body weight to prevent them from the stress of parasitism. Totally, 24 buffalo heifers above 2 years of age were selected and divided randomly in four equal groups (n=6). The herbs *A. marmelos* and *M. koenigii* were administered along with concentrate to the buffalo heifers for 9 days. In the present experiment, four group taken and Group I (n=6) treated with *A. marmelos*, Group II (n=6) treated with *M. koenigii*, Group III (n=6) treated with mixture of *A*. *marmelos* and *M. koenigii* and fed for 9 days. Group IV (n=6) considered as control and fed with concentrate only. The blood samples were collected from all the animals on day 0 (before treatment), 4, 9 (during treatment), on the day of estrus and day 8 after the onset of estrus. The 10 ml blood was collected from the jugular vein of all the experimental animals for estimation of serum calcium, inorganic phosphorus, and progesterone (P_4_). Feeding of herbal plant to heifer was done as described by Das *et al*. [2] and Dutt *et al*. [3] (Table-1).

**Table-1:** Treatment schedule.

Groups	Number of animals	Treatment
Group I	6	*A. marmelos*
Group II	6	*M. koenigii*
Group III	6	Mixture of *A. marmelos* and *M. koenigii*
Group IV	6	Control

*A. marmelos*=*Aegle marmelos*, *M.* *koenigii*=*Murraya koenigii*

### Preparation of herbal powder

The leaves were allowed for shade drying and subsequently placed in mixer grinder for making powder. The each herbal powder was packed in the separate plastic bag and kept at room temperature with proper labeling.

### Dose extrapolation from rats to heifers

Dose of extract for buffalo heifers was extrapolated from rats using the dose equivalent system through Km factor Van Miert [1]. The dose of extract was converted to the dose of leaves powder based on the basis of percentage yield using the following formula.


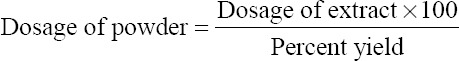


Leaf powder dose on per kg body weight basis was worked out for *A. marmelos* and *M. koenigii*, separately. For Group III animals, the dose of each plant was divided by two to calculate half of the dose and final dose was obtained by mixing both the herbs.

### Estimation of serum calcium, inorganic phosphorus, and progesterone assay

The estimation of macro minerals, namely, serum calcium and phosphorous was accomplished with the help of Semi Auto Analyser using commercial diagnostic kits manufactured by Avantor Performance Materials India Limited (Dehradun). The concentration of serum calcium and inorganic phosphorus was expressed in mg/dl, and progesterone concentration was also estimated by radio immunoassay technique manufactured by Immunotech, France (Bekman Coulter). The values were expressed in ng/ml.

### Estrus response and time interval for onset of estrus

All the heifers were observed for estrus on the basis of usual signs such as vulvar edema, cervical mucus discharge, stands to be mounted, bellowing, sniffing, and licking of vulva. The estrus response was expressed in percentage. The time required for onset of estrus was calculated from the initiation of treatment (day 1) up to the exhibition of the first sign of estrus, and it was expressed in days.

### Ovulation and conception rate

The buffalo heifers responded with estrus examined per rectally after 10 days for the presence of corpus luteum. The ovulation rate was calculated on the basis of the presence of corpus luteum. The heifers exhibited estrus was bred by natural service at appropriate time of estrus, and the pregnancy was confirmed after day 60. The ovulation and conception rate was expressed in percentage which was calculated on the basis of number of animals exhibited estrus.

### Statistical analysis

The data collected from various observations during the present study was analyzed by ANOVA – one-way as prescribed by Snedecor and Cochran [4].

## Result and Discussion

### Estrus response

From Group III (*A. marmelos* and *M. koenigii*) 4 heifers, from Group II 3 heifers (*M. koenigii*) and from Group I (*A. marmelos*) and IV (Control) 2 heifers each, exhibited the estrus (Table-2).

**Table-2:** Effect of herbal supplementation on estrus response and mean time interval recorded for onset of estrus in different groups.

Particulars	Group I	Group II	Group III	Group IV
Estrus response (%)	33.33	50.00	75.00	33.33
Time interval for onset of estrus (days)	19.50	16.33	14.50	26.50

The estrus response (33.33%) recorded in Group I buffalo heifers treated with *A. marmelos* was found to be lower when compared with the observation reported by Kumar *et al.*, [5] and they recorded onset of estrus within 10.0±2.00 days.

Satheshkumar and Punniamurthy [6] recorded higher the estrus response of 60.00% and 28.0±2.56 days in anestrus heifers of age 2-3 years when supplemented with dried leaves powder of *M. koenigii* which is slightly more as compared to the present observation.

In present experiment, estrus response of 75.00% was recorded in delayed pubertal buffalo heifers placed under Group III (*A. marmelos* and *M. koenigii*) not comparable with Das *et al.*, [7] as they reported 92.90% estrus induction rate in delayed pubertal buffalo heifers after supplementation of a similar combination of herbs for 9 days.

From the results, it is evident that the shade dried leaves powder of *A. marmelos* and *M. koenigii* combinely as well as *M. koenigii* alone found effective in induction of estrus in delayed pubertal buffalo heifers. The leaves are aromatic and contain proteins, carbohydrates, fiber, minerals like Mn, Cu, Fe, Zn, Co and carotene, vitamin C, vitamin A, calcium, and oxalic acid. The *in-vivo* and *in-vitro* studies have shown the antioxidant potential in *M. koenigii* leaves [8,9].

Recently, it has been reported that both plants promote the follicular development beyond 10 mm diameter in delayed pubertal heifers Kumar *et al*. [10]. In the present study, the active photochemical present in *A. marmelos* and *M. koenigii* might have manifested the stimulatory effect on follicular development, and therefore the estrus was exhibited by the buffalo heifers.

### Serum calcium and inorganic phosphorus

The mean serum calcium level recorded before and after treatment is presented in Table-3. The serum calcium level on day 0 and 4 was similar (p>0.05) among all groups. However, on day 9 and the day of estrus calcium level increased significantly in p<0.05 all treatment groups as compared to control group. In Group III, the value was significantly more (p<0.05) on day 8 post-estrus as compared to other groups. However, in treatment Group I significant increase (p<0.05) was noted on day 9 and the day of estrus. Similarly, in treatment Groups II and III, the serum calcium significantly increased on day 9, on the day of estrus and day 8 post-estrus. However, in control group, no significant difference was noticed in the level of calcium at any interval.

**Table-3:** Effect of herbal supplementation on mean serum calcium concentration (mg/dl) in various groups and intervals (ANOVA-one way).

Group	Interval			Estrus	Day 8^th^after estrus

	0^th^ day	4^th^ day	9^th^ day		
Group I	8.53^Aa^±0.63	8.39^Aa^±0.40	9.08^Ab^±0.20	8.85^Ab^±0.35	8.40^Aa^±0.20
Group II	8.52^Aa^±0.13	8.42^Aa^±0.21	8.81^BCac^±0.22	8.90^Bbc^±0.52	8.53^Aac^±0.32
Group III	8.65^Aa^±0.28	8.45^Aa^±0.44	8.94^BCac^±0.51	9.13^Cbc^±0.80	9.10^Bbc^±0.86
Group IV	8.58^Aa^±0.40	8.38^Aa^±0.39	8.47^Da^±0.41	8.52^Da^±0.75	8.40^ADa^±0.79

Mean values bearing different superscripts differ significantly (p<0.05) where a, b, c indicate difference within row and A, B, C, D within column

The mean inorganic phosphorus concentration recorded before and after treatment is presented in Table-4. Sampling within or between groups revealed no statistically significant difference (p>0.05) in the values recorded during pre-treatment, treatment, and post-treatment period of the experiment of sampling within or between groups.

**Table-4:** Effect of herbal supplementation on mean serum inorganic phosphorus concentration (mg/dl) in various groups and intervals (ANOVA-one way).

Groups	Interval			Estrus	Day 8^th^ after estrus

	0^th^ day	4^th^ day	9^th^ day		
Group I	7.44±0.99	7.94±0.56	7.35±0.90	7.58±0.83	7.63±0.68
Group II	5.76±0.47	5.74±0.68	7.60±0.86	7.83±0.80	6.93±0.32
Group III	6.98±0.56	6.92±0.85	6.80±0.57	6.51±0.68	6.73±0.38
Group IV	7.18±0.58	6.12±0.33	7.31±0.38	7.60±0.30	7.52±0.05

Kumawat *et al*. [11] recorded serum calcium concentration from 11.09 to 11.47 mg/dl in delayed pubertal heifers before treatment and observed significantly higher levels (p<0.05) on day 4, 6, 8, and 10 after feeding the dried leaves powder of *A. marmelos* and *M. koenigii* when compared with corresponding values of non-treated heifers. The findings corroborates with present observations.

Kumawat *et al.*, also investigated the effect of *A. marmelos* and *M. koenigii* on serum phosphorus of delayed pubertal heifers and observed that the level of phosphorus recorded from 4.86 to 5.31 mg/dl before treatment varied significantly (p<0.05) on day 2, 4, and 8 after the treatment when compared with the corresponding values of non-treated heifers. The findings do not corroborate with the present observation as no significant difference was found in the mean values of phosphorus between the groups and also at different intervals after treatment.

The present findings are in partial agreement with Das *et al.*, [8] as they reported serum calcium, phosphorus and Ca:P ratio as 11.78±2.41, 6.36±1.66 mg/dl, and 1.90:1, respectively in anestrus heifers supplemented with 100 g *M. koenigii* fresh leaves.

At hypothalamic level, calcium regulates the neuronal circuitry for gonadotropin-releasing hormone (GnRH) pulsatile secretion [12]. It is reported that calcium is involved in GnRH-stimulated luteinizing hormone (LH) and follicle-stimulating hormone (FSH) secretion from pituitary [13] and the deficiency of Ca or the presence of Ca blocking agents inhibits the FSH and LH release from the pituitary gland [14]. Calcium may also have a role in ovarian steroidogenesis [15].

The significant increase in serum calcium level on day 9, the day of estrus, and day 8 after onset of estrus in treated groups of the present study may be attributed to the *A. marmelos* and *M. koenigii*. During the present study, the phosphorus level before treatment was already recorded on higher side of normal range in all the groups which means that the delayed pubertal buffalo heifers selected for the present study were not deficient for inorganic phosphorus. Classical manifestations of phosphorus deficiency on reproductive processes involve alterations in estrus and decreased ovarian activity, anestrus, and delayed maturity [16].

### Serum progesterone concentration

In the present study, the progesterone concentration was estimated before and after the treatment, namely, on day “0” (before treatment), on day 4 and 9 (during treatment), on day of estrus and on day 8 after onset of estrus. The mean serum progesterone (P_4_) concentration recorded before and after treatment is presented in Table-5. Statistical analysis revealed no significant difference in the values recorded during pre-treatment, treatment period and on the day of estrus in all the groups and also at various intervals within group, however, highly significant (p<0.01) increase was noted on day 8 post-estrus in all the groups including control.

**Table-5:** Effect of herbal supplementation on serum progesterone (P4) concentration (ng/ml) in delayed pubertal buffalo heifers in various groups and intervals.

Group	Interval			Estrus	Day 8 after estrus

	0^th^ day	4^th^ day	9^th^ day		
Group I	0.21^a^±0.03	0.36^a^±0.14	0.37^a^±0.16	0.13^a^±0.01	3.40^b^±0.14
Group II	0.22^a^±0.03	0.34^a^±0.14	0.31^a^±0.13	0.16^a^±0.05	2.85^b^±0.36
Group III	0.23^a^±0.08	0.14^a^±0.02	0.14^a^±0.02	0.13^a^±0.01	2.60^b^±0.40
Group IV	0.21^a^±0.04	0.16^a^±0.02	0.18^a^±0.01	0.15^a^±0.01	3.15^b^±0.30

Mean values bearing different superscripts differ significantly (p<0.01)

a, b where indicate difference within row

Progesterone concentration estimated during the present experiment found to be in accordance with the findings of Dutt *et al.*, [3] and Kumawat *et al*. [3,11]. It is an essential ovarian steroid that regulates estrus cycle, maternal recognition of pregnancy and gestation in farm animals [17]. Apart from this, the hormone plays various important roles related to reproduction such as behavioral expression of estrus, ovulation, maintenance of uterine quiescence, and survival of the embryo and normal parturition [18].

From the present findings, it appears that the *A. marmelos* and *M. koenigii* do not have role in the production of progesterone. The significantly higher level of progesterone recorded on day 8 after estrus might have been due the presence of corpus luteum which is natural source of progesterone.

### Ovulation and conception rate

The conception and ovulation rate was given in Table-6. The conception rate recorded in Group I heifers treated with *A. marmelos* is much lower than the conception rate recorded by Kumar *et al.*, 2009 (100%) [5] with same drug, whereas the conception rate recorded for Group II heifers found to be slightly higher (66.67%) than reported by Shankar *et al.*, [19] (50.00%) in anestrus buffaloes treated with *M. koenigii* alone. Similarly, the conception rate calculated in Group III heifers (75.00%) found to be slightly higher but comparable with the observations recorded by Das *et al*. [2] and Dutt *et al*. [3] as they recorded 71.40% and 66.67% conception rate in delayed pubertal Vrindavani heifers and anestrus buffaloes, respectively treated with combination of *M. koenigii* and *A. marmelos*.

**Table-6:** Effect of herbal supplementation on ovulation and conception rate in delayed pubertal buffalo heifers after herbal treatment.

Particulars	Group I (%)	Group II (%)	Group III (%)	Group IV (%)
Ovulation rate	50.00	100	100	50.00
Conception rate	50.00	66.67	75.00	50.00

Results pertaining to the ovulation and conception rates are comparatively better in Group III buffalo heifers than heifers of other groups which may be attributed to the synergistic effect of *A. marmelos* and *M. koenigii*. The combined treatment of both herbs restored the fertility up to an appreciable extent. Furthermore, the conception rate recorded in Group II heifers also seems to be better as supplementation of *M. koenigii* alone resulted in more than 50.00% pregnancies. It may be inferred that as a result of plant treatment, gonadotropins from anterior pituitary or other mimicking active principles might have triggered the follicular dynamics by enhancing the follicular recruitment, selection, and preventing the atresia of follicles.

## Conclusion

Herbal supplementation of *A. marmelos* and *M. koenigii* in combination, as well as M. koenigii alone, were found effective in fertility improvement in delayed pubertal buffalo heifers. Herbal supplementation of *A. marmelos* and *M. koenigii* increases the serum calcium but do not alter serum inorganic phosphorus concentration and serum progesterone concentration. Thus, the herbal plants help enhancing fertility in delayed puberty buffaloes heifers.

## Authors’ Contributions

MMB collected a sample and processed as per standard protocol. APG design the work analyzed the data and help in making final manuscript. The UK helps in preparing outline research work and searching article. SKS and MSP helped in final proof of article. MansiMB helped in extraction of leaves. All authors read and approved the final manuscript.
